# The Impact of Sleep on Face Recognition Memory: A Scoping Review

**DOI:** 10.3390/brainsci12101385

**Published:** 2022-10-13

**Authors:** Isabel M. Santos, André Silva, Pedro Bem-Haja, Catarina Rosa, Luíza Cerri, Diâner F. Queiroz, Talles Barroso, Miguel F. Alves, Carlos F. Silva

**Affiliations:** 1 William James Center for Research, University of Aveiro, 3810-193 Aveiro, Portugal; 2 Department of Education and Psychology, University of Aveiro, 3810-193 Aveiro, Portugal; 3 ISEIT, Piaget Institute, 2805-059 Almada, Portugal; 4 CINTESIS@RISE, University of Aveiro, 3810-193 Aveiro, Portugal

**Keywords:** sleep, face recognition, memory, face identity, face learning, encoding, sleep stages, sleep restriction, sleep deprivation

## Abstract

Sleep has a major impact on a variety of human biological and cognitive functions. In particular, its impact on memory has attracted extensive research and has been amply demonstrated. However, it is still unclear whether sleep, or lack thereof, affects the ability to recognize faces. To clarify this, we conducted a scoping review on studies that included a face recognition memory task and any kind of sleep manipulation in adults without any sleep pathology. A systematic search and synthesis of peer-reviewed journal articles identified through the electronic databases Scopus, Web of Science, EBSCO, and PubMed was performed. A final sample of 18 articles, corresponding to 19 studies, met the eligibility criteria. The results of 13 articles suggested that sleep benefited face recognition ability, whereas two articles indicated a detrimental effect of sleep on performance, and four articles found no significant effects. This review highlights the high methodological variability between studies, in terms of sleep manipulation, retention interval, tasks used to probe face recognition, and other variables. In sum, although around one third of the studies show a beneficial effect of sleep on memory for faces, we suggest that future research should invest in replicating these findings with a stricter control of potentially confounding variables to allow stronger conclusions to be drawn.

## 1. Introduction

Intuitively, people understand the importance of sleep on a variety of human functions and variables. Indeed, research supports this notion, showing that sleep disturbances increase the risk of contracting illnesses (such as coronary artery disease [1]), and of developing high stress levels [2], hypertension [3,4], diabetes [5,6], infectious disease [7], neurocognitive impairment for a review see [8], and depression [9,10], leading to a higher mortality rate [11,12]. Some of these conditions may, in turn, exacerbate sleep disturbances creating further complications [13].

Sleep is also of the utmost importance to human cognitive functioning [14]. Studies with populations ranging from preschool children, e.g., [15], to adolescents [16] and older adults [17] all point to the detrimental effects of poor sleep on cognitive functioning, and in particular learning and recognition. Interestingly, several studies report an inverted U-shaped relationship between sleep duration and cognition, whereby a lack of sleep produces a decline in cognitive function but too much sleep can also produce a similar detrimental effect [18,19,20,21,22]. However, there is more to sleep than its duration. Studies show that sleep inertia, sleep homeostasis, and circadian phases all impact cognitive performance, and their relative influence depends on the specific cognitive function that is being assessed. For example, inhibitory control seems to be more affected by the circadian phase, while selective attention was more strongly impacted by sleep inertia [23]. Bernstein and colleagues [24] showed that greater sleep quality, measured by actigraphy, and longer sleep onset latencies were overall associated with better performance on measures related to conceptual flexibility, although there was an interplay between age and sleep quality, measured either objectively or subjectively, and cognition. In terms of memory, although evidence strongly suggests that sleep is highly relevant in the memory consolidation process, there is considerable debate regarding the impact of different sleep stages, as well as regarding the association between insomnia and memory deficits [25]. 

There also seems to be a relationship between sleep and emotional processing [26], in that a lack of sleep may increase emotional reactivity towards negative stimuli [27], which may be mediated by increased activity of the left amygdala [28,29,30]. On the other hand, some studies show that emotional reactivity is decreased in sleep-deprived individuals, such as when viewing facial expressions of both positive and negative valence [31,32]. Moreover, sleep deprivation has been shown to negatively impact one’s ability to experience both direct and indirect emotional empathy (i.e., to experience emotions while observing others) [33]. Even though the direction of the effect varies, research seems to demonstrate that sleep does influence reactivity to emotional stimuli and recognition of emotions. For an overview on the modulatory effects of various aspects of sleep on cognitive and emotional processes, see Walker [34].

### 1.1. On Sleep and Memory

Research relating sleep and memory goes a long way back. For example, already in the 1920s, the pioneering work of Jenkins and Dallenbach [35] showed that the rate of forgetting was more accelerated during wake time, compared to sleep. Sleep seems to improve the consolidation of emotional [36], declarative [37], and episodic memories [38] (for a review see [39]), even in children [40,41], and sleep loss seems to negatively impact emotion regulation [42]. Sleep deprivation also significantly impaired the recognition accuracy of emotional pictures, while both sleep deprivation and poor sleep quality resulted in a significantly more negative emotional valence attribution to previously seen stimuli [43]. Moreover, sleep seems to have a positive or facilitating effect on brain plasticity [44], relational memory [45], and insight ability for extracting implicit rules in learnt sequences [46]. Additionally, and despite some inconsistent results across studies and between objectively and subjectively reported cognitive difficulties in general [25], a meta-analysis revealed small to moderate impairments in episodic and working memory in individuals with insomnia [47], further reinforcing the important role of sleep in memory functioning. 

A considerable body of research has explored the relation between the various sleep stages and memory processes [48,49]. Sleep is organized in various stages, including the rapid eye movement (REM) stage, which is associated with dreaming, irregular muscle movements, reduced muscle tone, and rapid movements of the eyes, and three additional stages (N1, N2, and N3) which correspond to the non-REM (NREM) phase. N1 is a light sleep stage, whereas N2 and N3 are deeper sleep stages, with N3 being also known as slow-wave sleep (SWS), which is the deepest stage of sleep, with delta activity being predominant in electroencephalographic recordings [14]. 

Although the relation between NREM sleep and memory consolidation seems to have been more consistently established, there has been extensive debate regarding the involvement of REM sleep on memory consolidation and the exact nature of this putative relation [50,51,52]. Overall, it seems that declarative memory (including episodic and semantic memories) mainly benefits from NREM sleep, particularly N3-SWS, whereas non-declarative memory types (i.e., implicit, instrumental, and procedural memories) are more linked to REM sleep [14]. However, simple motor tasks, which involve only procedural motor learning, seem to be mostly affected by N2 sleep loss [53]. On the other hand, the formation of emotional memories might also benefit from REM sleep [54]. This differential impact of various sleep stages on memory has been termed “dual process hypothesis” [48]. Nonetheless, recent studies have focused more specifically on the role of N2 and SWS for both declarative and non-declarative memory. Evidence from these studies suggests that NREM sleep, besides its crucial importance for declarative memories, also seems to have a relevant role in procedural memory consolidation (sleep spindles in particular), with the role of REM sleep in memory processes still needing more clarification [48]. A recent study provided evidence that REM sleep seems especially relevant for memory refinement, which is related to how precisely the memory can be retrieved among competitive alternatives, whereas non-REM sleep can be associated with memory reinforcement, i.e., the ability to actually retrieve a memory [55].

### 1.2. On Sleep and Face Recognition: The Current Review

Face perception is of the utmost importance to our daily lives, and the ability to recognize someone’s identify is fundamental, either for social and personal reasons, or in various work contexts, such as security and forensic settings [56]. Faces provide some of the main and most reliable cues for person identification for models of face perception and recognition [57,58]. Additionally, faces provide cues to attractiveness [59,60], emotional status [61,62], and social traits and disposition [63,64], and thus are determinant in our social behavior. Face recognition seems to have a strong genetic basis and be highly heritable [65]. Additionally, there is evidence of numerous cognitive and neural specializations for face processing, including a distributed network of regions that show putative evidence of face-selective or specialized activity [58,66]. 

Face recognition depends on a particular type of memory that involves both explicit [67] and implicit [68] components. Given its important role on memory consolidation in general [69,70], sleep would also be expected to affect memory for faces and face recognition. Indeed, research seems to show that sleep, or lack thereof, seems to have an important impact on various aspects of face perception [32,71,72,73,74]. Those suffering from insomnia, for example, are shown to perform worse in recognizing certain facial emotions, such as fear [75] or anger [76]. A lack of sleep also appears to have a detrimental effect on recognizing face identity in a matching task that does not involve a memory component [72]. When we consider recognition memory for faces in specific, there is also evidence that sleep restriction negatively affects memory performance [77,78,79].

However, results are not consistent across studies, with some authors describing no effect of sleep deprivation on face recognition, e.g., [80], or even seemingly beneficial effects of lack of sleep, with short sleepers (less than seven hours per night) performing significantly better than average and long sleepers [81]. These apparently contradictory findings might be due to substantial methodological differences between studies. Thus, in order to draw clear conclusions regarding the effect of sleep on face recognition and how the underlying memory processes are being modulated, it is fundamental to identify the differences between studies and how those differences can influence the results that are observed. Given the heterogeneity of the research in this field, we carried out a present scoping review, which aimed to identify the available evidence in this area, examine how research was conducted, and systematize the main conclusions from the available studies, pointing to further directions and knowledge gaps that future studies should address [82]. To the best of our knowledge, no systematized summary exists on the effects of sleep on memory for faces.

Considering the objectives defined, through this scoping review we aimed at answering two main questions, centered around what the literature says about how the amount and/or quality of sleep influence our face recognition memory ability, as well as which methodological differences between studies might contribute to differences in study outcomes. It is important to note that the focus of our review will be the literature that explores effective behavioral performance in terms of face recognition memory, as we are interested in how sleep objectively affects this ability in adult participants, with fully developed face perception abilities. Therefore, we will not review the evidence collected by psychophysiological/imaging techniques that focus on the underlying neural mechanisms and how sleep modulates those processes. We will also not include studies involving participants with any kind of face recognition impairment or identified sleep pathologies, studies with infants or children, or studies where face processing was solely probed by indirect means (such as eye tracking).

## 2. Method

### 2.1. Search Strategy and Eligibility Criteria

Our review follows the Preferred Reporting Items for Systematic Reviews and Meta-Analyses Extension for Scoping Reviews (PRISMA-ScR) guidelines [83]. We conducted online searches in four databases: Scopus, Web of Science, EBSCO (all databases), and PubMed. All studies from first records through February 2022 were examined. Articles were selected if they: (a) included studies on human face recognition memory and any kind of sleep manipulation or variation in normal sleep patterns; (b) included adult participants, without any identified sleep pathologies; (c) were peer-reviewed journal articles; (d) were written in English or a language understood by at least one of the authors of this work (i.e., French, Spanish, or Portuguese). Literature/systematic reviews, book chapters, unpublished articles, commentaries, and conference abstracts were excluded. Searches in the databases were complemented by a manual search of the reference lists of included articles.

In particular, we employed the following string of keywords and Booleans: (sleep OR insomnia OR “sleep deprivation”) AND (“face perception” OR “facial perception” OR “face recognition” OR “facial recognition” OR “face processing” OR “facial processing” OR “person identification” OR “face identification” OR “face memory” OR “facial memory” OR “recognition memory” OR “face matching” OR “face learning” OR “facial learning” OR “facial matching”) AND (face* OR facial). Due to the specificities of each online database, small changes were made. On PubMed, our search was by title and abstracts; on Scopus, it was by title, abstract, and keywords; and on both Web of Science and EBSCO, the search was by topic.

### 2.2. Data Extraction and Synthesis Strategy

Titles and abstracts of all retrieved articles were screened by two of the authors (I.M.S., A.S.), and subsets of them were screened by each of the remaining authors. Doubts regarding the inclusion of certain articles were discussed by all authors. The full text of each selected article was read, and a final decision regarding their inclusion was made, also through discussion and consensus between the authors. The browser-based online computer application Rayyan [84] was used throughout this process to organize and screen records, and to make blinded decisions by each author.

From the selected articles, we extracted the following information: the sample information (the number of participants, gender, and age), the study design for the sleep-based manipulation and/or groups employed (e.g., sleep manipulation employing both an unrestricted sleep control group and a sleep deprivation group), the type of memory task used for face recognition (e.g., *n*-back, old-new recognition task), the training time of day (ToD) and recall ToD, the retention interval, whether and how prior sleep was controlled, the type of sleep manipulation control during the experiment, the sleep length, the main results, and the effect of sleep manipulation. Data collected from each study depended on the study design and tasks employed, and outcomes varied between the percentage of correct recognitions, false alarms, reaction times, or other measures of task performance (e.g., those based on signal detection theory, such as *d*′, bias, etc.).

## 3. Results

**Figure 1:**
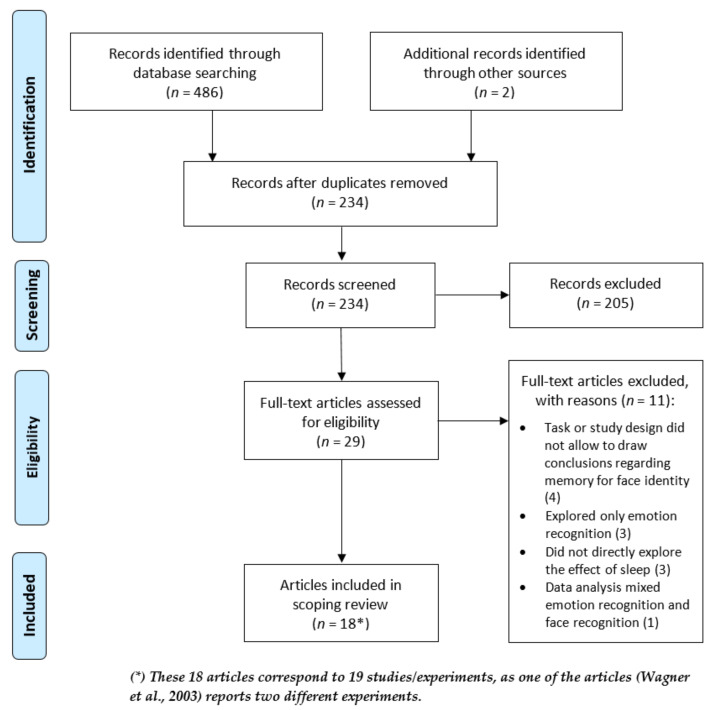
Flowchart depicting the selection process of studies for inclusion in the scoping review.

Our initial search yielded a total of 486 records. Two additional articles extracted through the snowball method described above were also included in the pool of articles to be reviewed. After removing all duplicates, a total of 234 records were retained and screened. After applying the inclusion criteria and all authors having resolved any conflicts, 205 articles were excluded based on their abstract. These were excluded for one or more of the following reasons: (a) were in a language not understood by any author of this work; (b) the sample comprised non-human animals; (c) the sample consisted only of infants, children, or adolescents; (d) the sample consisted only of participants with a clinical diagnosis; (e) the study employed a design which did not make it possible to determine the effect of sleep on face recognition; (f) were literature reviews; (g) were not peer-reviewed journal articles; (h) were related only to automatic machine facial recognition; (i) were not related to face recognition memory; (j) were related only to the recognition of emotional expressions in faces and not face identity recognition memory; (k) were not related to sleep or circadian rhythms; (l) it was not possible to retrieve the full text. Thus, we initially retained 29 articles for full-text reading. From these, we further excluded eleven articles after thoroughly reading the full text, mostly for using tasks that did not allow to draw conclusions regarding memory for face identity, exploring only emotion recognition, or not directly exploring the effect of sleep. Therefore, the final sample consisted of 18 articles to be included in the literature synthesis (see the flowchart of the literature search in Figure 1). These articles represent a total of 19 studies, as Wagner et al. [85] report two experiments.

A systematic description of these articles is presented below, including sample sizes and their characteristics, the study design and sleep manipulation and/or control group, the different face recognition tasks that were employed, and their main findings on the effect of sleep on face recognition. This information is systematized in Table 1 for each of the studies.

### 3.1. Sample and Demographic Characteristics

All retained articles employed non-clinical adult samples. On average, the sample size was 48.4 participants, ranging between 12 [79] and 182 [87], in a total of 920 participants across all studies and all experiments. As for gender, one article did not disclose this information [78], and the two studies of Wagner et al. [85] only included male participants. Studies in all other articles recruited both males and females (*n* = 16). Overall, and not considering the study that did not report gender, females accounted for 46.6% of all participants (*n*_total_ = 906, *n*_female_ = 422, *n*_male_ = 482). As for age, three studies did not report this information ([78], and the two studies of [85]), while three reported only an age range and not an average value [77,79,87]. For these, age varied between 18 and 39 years old. Of those studies which reported the mean age of their sample, the average was 23.3 years old. Most articles included a sample where participants were either controlled regarding past sleep habits or at least had them evaluated prior to participating in the experiment. Most of the articles had a control measure of the sleep manipulation during the experiment, either by having participants observed by the experimenter (e.g., in cases where sleep was restricted and the time was spent in the lab) or by more sophisticated means (e.g., polysomnography to control effective sleep or sleep stages). In some cases, the control was made simply by self-report (e.g., when the wakeful period took place during the day and participants were instructed to not take naps). One article did not report either measure, but gathered information about participants’ chronotype to control for the preferred time of day [89]. Chronotype was also assessed in some other studies, besides the sleep monitorization measures.

### 3.2. Experimental Design and Sleep Manipulation/Evaluation

Different authors employed different ways to control and/or manipulate sleep and wakefulness, both prior to participants’ arrival at the laboratory for the experimental sessions and during the experimental/retention interval. In general, studies either implemented some sort of sleep restriction during the common sleeping hours (i.e., participants were kept awake during all or part of the night), or the effect of sleep was studied by having participants simply sleeping or not sleeping before performing the task or during the interval between learning and testing, i.e., the retention interval (e.g., a 12 h interval between learning and testing) could occur between 8 am and 8 pm (normally not involving sleep) or between 8 p.m. and 8 a.m. (involving a normal sleep nigh), or participants could take a nap between learning and testing. Sleep manipulations could also be implemented between-subjects or within-subjects. Twelve studies employed a between-subjects sleep manipulation (63.1%), six employed a within-subjects design (31.6%), and one had a mixed design (5.3%) [85] in the main experiment. Among those employing a between-subjects manipulation, six compared a control group comprising participants with unrestricted or usual sleep durations with a group comprising participants with partial (*n* = 3) [87,94,95] or total sleep deprivation (*n* = 3) [88,90,97]. The remaining six studies compared groups with different sleep patterns or manipulations: short, average, and long sleepers [81]; with and without REM sleep deprivation [78]; with testing at different times-of-day and different retention intervals, with or without sleep, but with the absence of sleep occurring only during the daytime [89,96]; with or without a nap of one hour during retention [86]; or com-paring the effect of early (SWS) and late (REM) sleep between the study and the test (supplementary experiment of [85]).Among those studies which employed a within-subjects design (*n* = 6), half comprised a normal sleep and daytime wake [77,91,92], in which participants were tested after a full night of normal or unrestricted sleep and after a period during the day in which sleep was not allowed. In the remaining studies, participants were tested in two conditions, following a night of total sleep deprivation or normal/unrestricted sleep [80,93], or learning was followed by a night of sleep or by a full night awake, while testing occurred in the evening of the second day following a full night of sleep in both conditions [79]. Finally, in their first/main experiment, Wagner et al. [85] used a mixed design to implement two different sleep manipulations. On the one hand, they used a between-subjects manipulation to compare the effect of sleeping or not sleeping between the study and test phases. On the other hand, they used a within-subjects manipulation to explore the effects of SWS (first half of the night) vs. REM sleep (second half of the night) between the study and the test.

### 3.3. Face Recognition Task

Twelve of the nineteen studies included in this review (considering that [85] includes two experiments) employed an old-new identity recognition task to test memory for faces, where typically “old” faces (previously learnt) are shown among “new” faces (not seen before) and participants are required to indicate which faces they recognize and which are new, with occasional variations in the acquisition learning paradigm (*n* = 12, 63.2%). The remaining seven studies (*n* = 7, 36.8%) used diversified tasks and paradigms to test face recognition memory. Alberca-Reina and colleagues [94,95] employed a semantic-perceptual matching task in which participants were asked to learn pairs of faces who could share, or not share, the same profession, and later were asked to indicate whether two faces had previously been studied together or not. Frings [87] used a target detection task, where two faces were initially shown and had to subsequently be identified among several quartets of faces, either target-present or target-absent. Hussain and collaborators [89] employed a face identification task where a face that was presented at the beginning of each trial had to be identified amongst an array of 10 simultaneously presented faces. Martella and collaborators [90] also used a face identification task where, in each trial, an array of six faces was presented simultaneously, followed by a short interval, and afterwards a single face was shown and participants had to decide whether it had been present in the previous array or not. Maurer and colleagues [91] employed a face-name task, where participants learnt to associate names to faces and then were required to indicate if a specific pairing was correct or incorrect. Finally, in their main experiment, Wagner and collaborators [85] used a repetition priming task. During the study, participants were asked to indicate the sex of each face; during test, where the same faces were presented among distractors, participants were asked to indicate the viewing direction of the faces; face recognition would be inferred by the priming effect that “old” faces were expected to have on the viewing direction decision, thus being an implicit task.

In most of the studies, the old-new recognition tasks were explicit, i.e., participants knew that their recognition memory was going to be assessed beforehand. However, in some studies, recognition was implicit, e.g., in [85]. In the main experiment, no explicit memorization instruction was given, e.g., in [79], or this information was not explicit in the articles’ methods description.

### 3.4. Retention Interval and Time of Day of Learning and Testing

The retention interval, i.e., the time between learning and encoding and between testing and retrieval, is a key factor in analyzing memory effects and face recognition; thus, we also investigated this metric. Retention intervals varied substantially among the studies included in this review. Some studies used immediate recognition tasks or very short intervals, equal to or shorter than 15 min ([80,87,88,89,90], two of the groups in [97], and two of the experimental conditions in [96]). It is of note that most of these studies used a pre-task sleep manipulation, i.e., groups or conditions differed in the amount of sleep they had before coming to the lab to perform the task. Other studies used relatively short intervals, during which sleep could have (or could have not) occurred, and these were post-encoding sleep manipulations, i.e., sleep/no sleep took place after the learning/training session. Barton and Pietrowski [86] had a 1 h retention interval, and both experiments of Wagner and colleagues [85] reported a 3 h retention interval, while the retention interval used by Solomonova and collaborators [78] was between 6 and 7 h. Other studies reported retention intervals between 12 and 24 h, during which the sleep manipulation took place (as was the case in [77,81,94,95], in two of the groups in [97], and in four of the experimental conditions in [91,92,93,96]). Finally, the retention interval could also be longer than 24 h, with Wagner and colleagues [79] reporting a retention interval of 43.5 h, given that learning always took place in the evening immediately before the sleep or wake night, while recognition took place in the evening two days later, and one of the conditions of Sheth and colleagues [96] used a retention interval of 36 h, with two nights of sleep between learning and testing. It is important to note that, in some studies, the retention interval was not the same between the experimental conditions, which could introduce a confounding variable. For example, Mograss and collaborators [92] used a retention interval of approximately 15 h in the sleep condition, whereas in the no-sleep condition, the retention interval was only 9 h. A similar discrepancy occurred in Mograss et al. [77], although with a smaller difference between conditions (approximately 6 h). 

Because the time of day is a relevant variable in cognitive performance [98,99], the time when learning and testing took place is also a relevant aspect. Again, there was significant variability between studies regarding this variable, and also differences between conditions of the same study. Among studies involving a pre-encoding sleep manipulation, for example, in the study by Chee et al. [80], testing in the sleep condition took place at 8 am, whereas in the restricted sleep condition, testing took place at 5 am. In the study by Frings [87], participants in the sleep condition performed the task at 7 pm, having slept the night before, whereas participants in the restricted sleep condition performed the task at 10/11 am. Martella et al. [90] presented a similar distribution of times, although sleep and no-sleep testing hours were 8 pm and 4 am, respectively. In Harrison and Horne [88], participants in both normal sleep and total sleep deprivation conditions were all tested between 7:30 and 8:30 pm. In studies which used a post-encoding sleep manipulation, there was equal variability, as some studies performed the training in the evening and the retrieval in the following morning for the sleep condition, and the opposite was carried out for the no-sleep condition (i.e., encoding in the morning and testing in the evening), e.g., in [77,91,92], whereas others carried out the study in the evening and retrieval in the morning, e.g., in [78,93], or both learning and testing in the evening [79] for both normal sleep and sleep deprivation conditions. Interestingly, two studies attempted to disentangle the effects of sleep (pre- or post-learning), the retention interval, and the time of day when the tasks were performed with various experimental conditions that manipulated these variables, albeit in different ways and not in a fully counterbalanced mode [89,96].

### 3.5. The Effects of Sleep on Face Recognition—Summary of Evidence

Thirteen of the studies considered in this review found significant effects of sleep on recognition (68.4%), albeit some of these showed only conditional effects, i.e., significant effects dependent on certain conditions or after certain manipulations. Only two studies (10.5%) presented results that suggest a detrimental effect of sleep on face recognition memory. The remaining four studies (21.1%) found non-significant effects of sleep on recognition.

Considering the thirteen studies which did find positive effects of sleep, the results were still quite variable. In summary, the following was observed: there was no effect of sleep deprivation on accuracy but a significant effect of participants’ performance on the temporal memory aspect of the test (there was a recency effect in which sleep-deprived participants were less able to determine whether the stimuli were presented in the first or second set of stimuli [88]); there was a small positive effect of sleep on recognition but conditional to the first trials of the second session [89]; there was no direct effect of sleep on face memory, but a positive correlation between N3 sleep duration and negative face recognition, which suggested more elaborate processing during SWS, resulting in better memory for negative valence faces [86]; there was a disruption of face recognition ability due to sleep loss that was detected in a response bias towards identifying the target as being absent [87]; there was a decreased percentage of correct responses in participants with total sleep deprivation compared with controls with unrestricted sleep [90]; there was better performance after a night of sleep compared with daytime wake without sleep for the same participants [77,91,92]; there was worse performance when a night of total sleep deprivation followed learning, in comparison to a night of normal sleep, also in within-participants [79,93]; there was a decreased percentage of correct responses for REM-deprived participants compared to non-REM-deprived participants [78]; and, finally, there was a positive effect of REM sleep on memory, strengthening automatic face recognition, which interfered with performance on an implicit priming task (this effect was clear in the 2003 supplementary study of Wagner and colleagues [85], which then explained the apparently contradictory results of the main study). 

Although a large number of studies used an old-new recognition task to test face memory (the basic/classic version) or introduced some modifications (particularly at learning), other differences in methodology were identified, such as retention interval, sleep manipulation, and study design, which may have impacted the results and conclusions.

### 3.6. The Effects of Sleep on Face Recognition—Detailed Findings

The various studies, as well as their main methodological characteristics and results, will be summarized below, and then organized according to the effect of sleep on face recognition memory (positive effects, negative effects, and null effects). Please note that we consider “positive effects” those where the presence of sleep benefited performance, in comparison with sleep restriction conditions, and “negative effects” as those where more sleep seems to impair performance. It is important to highlight that, in studies with additional objectives besides exploring how a sleep manipulation impacts face recognition, we will focus exclusively on the results pertaining the effects of sleep on face memory. Again, more details can be found in Table 1.

#### 3.6.1. Positive Effects, i.e., Beneficial Effects of Sleep on Performance

Chronologically, the first article reporting positive results of sleep on memory for faces included in the present review is that of Harrison and Horne [88]. Although the authors concluded that there was no effect of sleep deprivation on face recognition accuracy (*d*’), there was an effect of sleep on recency discrimination, as sleep-deprived participants showed impaired temporal memory in face recognition and were less able to indicate the source list of the face stimuli. Participants in the sleep-deprived conditions arrived at the lab early in the morning on the first day and remained there until the evening of the next day, while those in the non-sleep-deprived groups were allowed to go home in the evening and return the next day. The time of day was kept constant as the task was performed in the same time period (evening) for all participants. The task involved a presentation of two sequences of 12 color photographs of faces for 10 s each, with each of them being followed by a five-minute filler task. Afterwards, an old-new recognition task followed, whereby a follow-up question was added whenever participants responded they recognized a face, asking them to indicate whether that face was included in the first or second set, allowing the authors to assess recency discrimination and thus temporal memory. Participants were also asked to rate their confidence on whether their response to the recency question was correct. Although no significant group effects were found on face recognition accuracy, sleep-deprived participants were significantly worse on the temporal memory assessment. There was also a significant group-accuracy interaction, whereby sleep-deprived participants were more confident that their answers were correct especially when they were wrong. Thus, sleep deprivation seems to have impaired temporal memory for faces, although not recognition per se.

In their main experiment, Wagner and colleagues [85] were interested in studying the effect of sleeping or not sleeping during a retention interval of 3 h on two night periods: an earlier period dominated by slow-wave sleep (SWS) and a later period with pronounced REM sleep. The retention period (early vs. late) was manipulated within subjects, and the sleep vs. wake during retention was manipulated between subjects, in a mixed design. Their main hypothesis was that REM sleep during the retention interval would enhance implicit memory for faces; thus, it used a repetition priming task to test it (see details of the task in Table 1). The results, however, apparently contradicted the hypothesis, showing an inverse priming effect, with longer reaction times for judging the viewing direction of previously seen faces in the late-sleep condition, seemingly indicating that REM sleep impaired face recognition memory. The authors hypothesized that this could in fact result from enhanced automatic face recognition, which interfered with, and caused a delay in, responses in the implicit priming task. Therefore, Wagner et al. [85] conducted a supplementary experiment, manipulating only the early- vs. late-sleep period between subjects, and used an explicit old-new face recognition task for the test. This experiment confirmed the predictions, showing a positive priming effect with a faster RT for old faces, which was more pronounced in the late-sleep condition, suggesting the beneficial effect of REM sleep on the consolidation of memory for faces.

Mograss and collaborators [77] applied a classical old-new recognition task with unfamiliar faces and explicit instructions to memorize the stimuli. There could be a night of sleep or daytime wake between acquisition and recall, employing a within-subjects design. There were differences in the time of day for both acquisition and recall: in the sleep condition, acquisition occurred in the evening and recall occurred in the morning, whereas in the daytime wake condition, it was the opposite. They found a significant difference in the percentage of hits (i.e., the number of correct recognitions of old items) between the sleep and wake conditions, with performance improving when sleep occurred during retention, thus suggesting a positive role of sleep in memory consolidation.

Wagner and colleagues [79] reported similar results in a study using emotional faces (displaying happiness, anger, or a neutral expression) to investigate whether sleep enhanced general face recognition. In their study, using a within-subjects design, the time of day for learning and recalling was kept constant across sleep and wake conditions. Sleep was either allowed or not in the night after encoding, and testing took place two evenings later, allowing a full night of sleep before testing in both conditions (around 43 h retention interval). The memory task was a classical old-new recognition task. The results showed that memory accuracy (i.e., hit rate minus false alarm rate) was higher in the sleep condition than in the wake (i.e., sleep-deprived) condition, indicating that sleep after learning led to better memory performance. This was independent of facial expression. Additionally, in the sleep condition, significant positive correlations were observed between the overall memory accuracy and the non-REM sleep and total sleep duration (but not REM sleep) in the night following the learning session (i.e., the consolidation sleep).

Using a within-subjects manipulation of sleep vs. wakefulness and a classic old-new face recognition task, Mograss and colleagues [92] also reported positive effects of sleep in face recognition memory. They attempted to control for circadian influences on performance, by conducting the learning and testing in both conditions during similar time windows. In the sleep condition, learning occurred in the evening and testing was in the morning, while the opposite occurred in the wake condition. The results showed a marginally higher hit rate (the correct recognition of “old” items) and significantly fewer misses (old items failed to be identified) in the sleep condition compared to the wake condition. Altogether, this suggests a beneficial role of sleep in the consolidation of memory for faces. Since no differences were found for response times (RTs), the authors suggest that sleep does not affect the quantity of information retrieved or the complexity of the process (indexed by RT). Instead, it is the quality of the memory trace or the accessibility of the information in memory (which is indexed by the accuracy of performance) that is positively affected by sleep.

Hussain and colleagues [89] aimed to investigate the effect of sleep and time intervals between sessions on the overall performance improvement (learning) on a face identification task. In a between-subjects design, the manipulated variables were time-elapsed between sessions (12 h vs. 24 h vs. 3 h), sleep (sleep vs. no-sleep), and time of day when the task was performed (9 a.m. vs. 9 p.m.), originating in five groups (see Table 1 for times of testing). The task consisted of many trials presenting a single face, and immediately after each presentation, participants were required to recognize that face among an array of 10 faces with different levels of noise and contrast. The proportion of correct responses did not differ between groups in either session, with overall better performance in the second session. However, of interest to this review, the authors reported a trend towards an improvement from the first to the second session for the sleep compared to the no-sleep groups (which nonetheless failed to reach statistical significance, *p* = 0.052), and, among those who slept, significantly greater learning was observed for those with a 12 h interval between sessions compared to those with a 24 h interval, suggesting that the benefits of sleep were greater for the shortest between-session interval. In addition, the authors also analyzed their data in terms of the time course of within-session learning by creating several bins of trials, showing an improvement in performance attributed to sleep, but only for the first bin (105 trials) of the second session. Despite the positive differences found, the authors concluded that the effect of sleep on between-session learning was negligible and that “robust perceptual learning for a face identification task can be obtained in the absence of sleep” (p. 2792).

Mograss and colleagues [93] aimed to explore the impact of one night of total sleep deprivation (TSD), compared to one night of normal sleep on memory for unfamiliar faces with neutral expressions again under a classical old-new paradigm, maintaining the same learning (4–6 p.m.) and testing hours (7–9 a.m.), as well as a within-subjects design. Although no effects of sleep deprivation were observed on the percentage of correct responses to “old” items, the authors reported a trend for the correct rejections of new stimuli (which did not reach statistical significance, *p* = 0.07), with worse performance after TSD than after a night of sleep. Additionally, responses were significantly slower on false alarms (when “new” items are incorrectly judged as being “old”) in the TSD condition than in the sleep condition. Altogether, these results suggest an increased difficulty in discriminating new from old stimuli after sleep deprivation.

Martella and colleagues [90] conducted an experiment in which two groups were compared: a group of sleep-deprived participants (performing the task at 4 a.m., without any sleep) and a control group with an unrestricted regular sleep schedule (performing the task at 8 p.m.), denoting different testing times for both groups. While their main goal was to assess whether the differential outcomes procedure (DOP) could enhance the memories of participants that were sleep-deprived, they reported main findings on the effect of sleep deprivation on face recognition memory. The retention interval between the sample stimuli (a matrix of six faces) and the probe faces (to be recognized) was a short interval variable between 5 and 32 s. The results showed that sleep-deprived participants displayed a significantly lower percentage of correct responses, and a shorter recall delay led to a higher percentage of correct responses only in the control group. Interestingly, at the chance level, sleep-deprived participants performed both short and long delays, suggesting that the task was significantly harder under conditions of sleep deprivation.

Maurer and colleagues [91] used a face-name association task where participants were presented with photographs of faces paired with a name, and asked to memorize the name associated with each face. For the recognition phase, seen faces were presented with the same or different names and participants were asked to indicate whether the pairing was correct or not. They also provided confidence ratings on a nine-point Likert-type scale. The interval between learning and recognition was 12 h, and participants completed the task twice, in the “sleep” and “wake” conditions, counterbalanced. Of importance, the time of day was different between conditions. In the sleep condition, the learning phase took place in the evening and the recognition phase took place in the morning, whereas it was the opposite in the wake condition. The results showed that the number of correct responses was greater in the sleep condition compared to the wake condition. In addition, sleep between learning and recognition resulted in 12% more highly confident responses (ratings of seven or above) and 30% less incorrect responses. Additional analysis to examine the possible effects of time of day in encoding and recognition yielded no relevant results, arguing against that possibility.

Frings [87] used a target identification task in a four-face line-up to investigate the effect of fatigue (experimentally introduced through partial sleep deprivation) and team membership (not explored here) on face recognition, using a between-subjects design. Although the experimental manipulation did not entail complete sleep deprivation, it sheds some light on the effects of even relatively minor sleep loss, which is compatible with real-life patterns. Face recognition sessions took place around 7 p.m. for participants in the rested/sleep condition, and between 10 and 11 a.m. after two days of intense physical and mental activity, and two sequential nights of sleep loss (sleep duration was limited to five hours and sleep was systematically disturbed by being forced to spend one hour awake in a watch duty activity) for participants in the fatigued condition. The task consisted of a sequence of trials where participants were shown a pair of faces and immediately after were asked to indicate whether either of those target faces was present or absent on a four-face line-up. The data were analyzed using the indexes of the signal detection theory. Interestingly, although no effect of fatigue was observed for hits, false alarms, or detection accuracy (*A*′), such an effect was evident for response bias. Sleep-disrupted participants had a higher response bias (*B*″) than participants in the rested/alert condition, indicating a stronger tendency to identify a target as absent (i.e., missing its recognition). In light of these results, the authors point out that it is important to consider various measures of performance, because some might be more sensitive to the effects of fatigue than others.

In the study of Solomonova and colleagues [78], all participants spent one night sleeping at the lab and were randomly assigned to one of two groups: a control group or a “partial REM sleep deprivation group” (REMD). Although all participants had their sleep interrupted regularly, the control group underwent awakenings after 25 min of REM sleep had elapsed, while the REMD group was always awoken after five minutes of REM sleep onset. Prior to this sleep manipulation, all participants were required to perform a virtual reality task, where they had to navigate a village and engage in face-to-face pre-determined conversations with 10 computer-generated avatars whose facial characteristics were unique. In the morning, participants were asked to perform an old-new recognition task for all previously engaged virtual characters’ faces plus distractors. The results showed a significantly higher proportion of correct responses in the control group compared to the REMD group. The authors conclude that the time spent in REM sleep might be especially important in consolidation of memory for faces. Additionally, sleep spindles might also play a role in this process, although this is not yet fully understood. 

The last study showing positive results of sleep on recognition in our review is that of Barton and Pietrowsky [86]. The authors manipulated sleep between subjects with two groups: the sleep and wake groups. The task was a classical old-new recognition paradigm after explicit memorizing instructions at encoding, with emotional faces (positive, negative, and neutral valence). The learning phase took place either at midday or early afternoon, after which a one-hour retention interval followed, with testing occurring between 1 and 4 pm. Participants in the sleep group slept during this interval, while those in the wake condition watched television. After this period, all participants performed an old-new face recognition task. The results showed no effects of sleep and no interaction between sleep and valence on memory accuracy, response bias, and response times, leading to the conclusion that a nap during the retention interval did not improve facial recognition. Nonetheless, there was a significant positive correlation between the duration of the N3 sleep stage and recognition memory for negative valence faces, which the authors interpreted as suggesting a beneficial effect of slow-wave sleep (which predominates in N3) on memory consolidation, which is selective for negative emotions.

#### 3.6.2. Negative Effects, i.e., Detrimental Effects of Sleep on Performance

Contrary to the studies described above, the results reported by Mograss and colleagues [81] suggested a potential detrimental effect of sleep on face recognition. This study has the particularity of having recruited participants according to their habitual sleep duration and not introducing an experimental manipulation of sleep. Accordingly, participants were separated into short sleepers (those who normally slept less than seven hours per night), average sleepers (those who normally slept between seven and nine hours per night), and long sleepers (those who normally slept more than nine hours per night). The experimental task consisted of a classical old-new explicit recognition task, with the learning phase taking place in the evening and the testing session in the next morning after a night of sleep in the lab. Importantly, on average, participants retained their group’s habitual sleeping hours. The results showed that short sleepers produced a significantly higher percentage of correct responses to old items compared to both average and long sleepers. No difference was found between average and long sleepers. Of further interest, a significant negative correlation between percentage of correct responses and number of hours of sleep was observed for short sleepers, while for average and long sleepers, this relation was reversed, with increased accuracy being associated with significantly longer sleeping hours. It should be noted that this study did not compare sleepers with sleep-deprived participants, so the effect of sleep is really an effect of the magnitude of sleeping hours on recognition and not necessarily of sleep itself. Besides this putatively detrimental effect of longer sleeping hours in recognition memory, it draws attention to the potential importance of individual differences in sleeping patterns on face memory performance.

Alberca-Reina and colleagues [94] developed a study that aimed to understand the effect of sleep restriction on memory encoding and consolidation for pairs of faces that could be either semantically congruent (i.e., shared the same profession) or semantically incongruent (i.e., had different professions). Sleep restriction (allowing only four hours of sleep) was imposed on the night before the encoding session to a group of subjects, whereas another group suffered a similar sleep restriction on the night after encoding (i.e., consolidation period), and the control group was allowed eight hours of sleep on both nights. On the morning after the encoding session, participants were tested for their associative memory for the original face pairs after an interference task. All groups were allowed a full night of sleep (recovery sleep) on a second night after encoding, and in the following morning, there was a second testing session, this time without interference. Despite the complex paradigm, results seemed to indicate that, in the second testing session, in comparison with the control group, the pre-training sleep restriction group showed enhanced recognition for semantically congruent faces and decreased recognition for semantically incongruent faces; on the other hand, the post-training sleep restriction group showed a trend towards enhanced memory for both semantically congruent and incongruent associations, also in comparison to the control group. Although the main focus of the work was on the interaction between sleep loss and semantic congruency effects on memory, for the purpose of this review, the results seem to suggest that sleep restriction exerted a beneficial effect on associative recognition memory for face pairs.

#### 3.6.3. Null Effects, i.e., Sleep Manipulation Has No Effect on Performance

As mentioned above, some articles included in this review showed no effects of sleep on recognition. A summary of each will follow.

Sheth et al. [96] attempted to disentangle the effects of sleep from the effects of time spent awake and time of day, in a study with a large number of subject groups, also using a simple old-new face recognition paradigm, but this time with computer-generated faces. They did so by creating seven randomly assigned participant groups where time-of-day for acquisition and testing, retention interval, intervening sleep, and intervening wake were systematically varied (see Table 1 for details). Contrary to their predictions, no beneficial effect of sleep was found for the strength of recognition memory (assessed with *d*′) or for how liberal/conservative the response bias was. Instead, a longer duration of wakefulness between learning and testing was found to negatively impact recognition memory strength and to render the participants’ responses more conservative, i.e., they were less likely to indicate that they had seen a test face before (regardless of being an old or new face). The authors claim that their results strongly advocate for a negative effect of the interference of ongoing visual stimulation (e.g., other faces encountered) on memory retention during wakefulness, instead of a positive effect of sleep on face recognition memory.

The work presented by Chee and colleagues [80] aimed to compare the effects of one night of sleep deprivation (SD) with rested wakefulness (RW) on object-selective attention, under a repeated measures design, and also used an old-new recognition paradigm. During learning, participants were exposed to alternating sequences of grayscale images of neutral faces and outdoor scenes and were required to attend either to the faces or the scenes (while ignoring the other), or attend both, and indicate whether it was a male or a female (attend faces or both) or whether the image contained water (attend scenes or both) for each relevant image in each condition. The recognition of these items among distractors took place around 15 min after learning, and the sessions occurred at 8 am (after a full night of sleep—the RW condition) or 5 a.m. (after a nigh awake—the SD condition). The results showed that sleep deprivation led to a greater number of invalid responses across conditions during the learning phase (responding to non-target stimuli that should be ignored or failing to respond to target stimuli), and that participants, overall, were slower and had a less accurate performance under SD. However, when looking at memory performance during testing, sleep deprivation decreased recognition only for scenes, while recognition of faces was not impaired. The authors suggested that this may be because face recognition might be a more automatic process, but do not discuss it further. 

Alberca-Reina and colleagues published a second study in 2015 [95] using a similar paradigm to the one of the 2014 study, again looking at the effects of partial sleep restriction on memory for pairs of faces. In this study, there were only two groups: a control group that had a full night of sleep and an acute sleep restriction group that slept only 4 h on the night pre-training. Training then occurred at 6:30 p.m. and after one night of recovery sleep, participants were tested at 12:00 p.m. the following day, after an interference task (9:30 a.m.) [for more details regarding the tasks, please see the description of the study of 88 above]. The results indicate that there were no significant differences between the control and acute sleep restriction groups in any memory indices and there were no interactions between the group and the semantic congruence of the face pairs. Thus, we can conclude that there were no effects of sleep restriction on behavioral task performance regarding recognition memory for face-face associations.

Finally, the most recent work to show no effect of sleep on performance in our review is that of Stare and colleagues [97]. The authors attempted to explore the effect of post-learning sleep compared to wakefulness on memory for incidentally learned faces. Using a between-subjects design, they had two experimental groups, which either slept (learning in the evening and testing in the morning) or remained awake (learning in the morning and testing in the evening) during a retention interval of approximately 12 h, and two control groups, which performed the task (learning and immediate testing) either in the morning or in the evening to control for circadian influences on performance. During the encoding phase, participants indicated whether each person/face was likely to help them answer a specific question that was presented immediately before. During the testing phase, the previously seen faces were shown among new faces, and participants were asked to rate their confidence on a 1–4 scale on whether they recognized each face. The results showed no main group effects or interactions with the group, indicating no effect of post-learning sleep on memory for faces.

## 4. Discussion

It is well established that sleep can have a major impact on various areas of cognitive function and emotional processing [14,34]. In particular, the important role of sleep in memory consolidation has been demonstrated [39]. Although there is evidence that sleep restriction seems to negatively affect memory for faces [93], there is some inconsistency between studies which may be due to diverse methodological differences. To the best of our knowledge, the literature on this topic has not been systematized before. Therefore, this scoping review aimed to identify the studies that focused on the effects of sleep on face recognition memory, systematize their results and identify the methodological factors that might introduce noise in the conclusions that can be drawn and might justify different outcomes. We focused our attention on the effect of sleep on behavioral performance in healthy adults.

After the application of all eligibility criteria, we selected 18 articles that corresponded to 19 studies. From these studies, thirteen reported positive effects of sleep on face recognition memory, as recognition performance was either impaired after some sort of sleep restriction, or performance was better after sleep than after an equivalent time spent awake. Some studies also demonstrated specific effects for different sleep stages. Solomonova et al. [78] reported the detrimental effect of REM sleep deprivation on recognition performance, while Wagner et al. [85] concluded that REM sleep seems to strengthen automatic face recognition. Barton and Pietrowski [86] found a positive correlation between N3 sleep and the recognition of faces expressing negative emotion, and suggested that more elaborate processing occurred during this sleep stage. While four studies found no significant effects of sleep on face recognition, two studies actually suggested that sleep after encoding or longer sleeping habits might impair subsequent face memory [81,94]. Although the majority of the selected studies found beneficial effects of sleep on face recognition performance, which is in agreement with the literature which demonstrates the importance of sleep for memory functioning and the damaging effects of sleep loss on memory [69,100], studies still varied considerably in various methodological aspects, which are important to highlight. 

Thus, there were considerable differences in the study design used for sleep manipulation (between- or within-subjects); when the sleep restriction was introduced (before encoding or before task performance when recollection was immediate, or after encoding but pre-testing, i.e., during the retention interval); how long the retention interval was (varying from immediate recognition to a couple of days); the time of day when the training and testing took place (morning or evening, for example), and whether these times of day varied between conditions where sleep was allowed or restricted, as well as the time of day when the retention interval occurred (i.e., during the night or during the day); whether there was total sleep deprivation (i.e., participants spent a full night awake) or only sleep restriction or disruption (where only some hours of sleep were allowed, or sleep was systematically disrupted with periodic awakenings, sometimes to inhibit specific sleep stages); the actual task that was used to probe face recognition memory, which could vary both in the encoding protocol, or how memory was tested (the most common being the classical old-new recognition paradigm, although several variations could occur), or even in the type of stimuli that were used (real face photographs, computer-generated faces, or 3D characters); and whether there were explicit instructions regarding memorization of the face stimuli or whether learning was incidental.

Studies also differed substantially in the dependent variables that were used to assess recognition memory. These varied between the direct analysis of percentage or the proportion of correct responses, hits, false alarms, misses, and correct rejections, but also measures of signal detection theory, such as *d*′/*A*′ and response bias. Other studies also analyzed response times. Thus, it might sometimes be hard to draw comparable conclusions based on considerably different metrics, particularly because sometimes sleep effects are found for some metrics but not for others [87,96], making it harder to interpret and compare results across studies. 

In several studies, the research focus was not on the main effect of sleep on performance. Instead, researchers were interested in exploring the interactions between the sleep manipulation and other variables, i.e., how sleep restriction modulated the effect of other variables unrelated to sleep patterns. These could differ considerably between studies and often increased the difficulty of interpreting sleep effects. For example, Wagner et al. [79] aimed to understand how sleep or wakefulness after learning affected recognition memory for faces with different emotional expressions. Martella et al. [90] were interested in exploring whether reinforcing correct responses would minimize the negative effects of sleep deprivation on memory performance. Alberca-Reina and colleagues [94,95] examined how the congruency of newly learned information with prior knowledge interacted with the effects of sleep restriction before or after encoding on associative memory for pairs of faces. In these studies, one of them demonstrated a seemingly detrimental effect of sleep on memory performance [94], and the interpretation of sleep effects was indeed rather convoluted due to the large number of variables included in the study and the interactions between them. 

From the 19 studies included in this review, 2 of them presented evidence in favor of a potentially disadvantageous effect of sleep, compared to sleep restriction, or habitually long or average sleeping hours, compared to short sleeping hours, on memory for facial stimuli [81,94]. It is worth examining these studies in a little more detail to try and understand these effects. The study of Mograss et al. [81] comes after a sequence of studies that showed positive effects of sleep on memory for faces [77,92,93]. All those studies used a within-subjects sleep manipulation, with participants either sleeping or staying awake during the retention interval. In the fourth study, Mograss and collaborators [81] opted to manipulate sleep by exploring individual differences in sleep habits (between-subjects manipulation) and recruiting three groups of participants that differed in their regular sleeping hours (short, average, and long sleepers). The results showed that short sleepers had better memory performance than average and long sleepers, which did not differ. In addition, in short sleepers, the number of hours of sleep was negatively correlated with performance. However, in average and long sleepers, this correlation was positive. Two things are worth pointing out regarding this study. First, it suggests that individual differences seem to be a relevant variable to take sleep-related studies into consideration, and the mechanisms underlying the performance of short sleepers should be explored further. Second, the direction of the correlation observed for average and long sleepers actually goes in a direction that is consistent with studies reporting a positive effect of sleep on memory performance. As the prevalence of short and long sleepers in the general population is lower than that of average sleepers [101], and as most other studies generally recruit only average sleepers with regular sleeping habits, the results are in fact compatible. Thus, it is the short sleeper’s performance that stands out in Mograss et al. [81] and should be worthy of further exploration and replication.

Regarding the second study that provided evidence for an adverse effect of sleep on memory performance [94], as mentioned above, it involved a complex experimental protocol. It included different sleep manipulations (sleep restriction pre- and post-training) together with other variables, such as the semantic congruence of the pairs of faces that should be memorized, and the existence of an interference task. The tasks also tested directly associative memory and not memory for individual stimuli, which represents a substantial change from other studies. The significant positive effect of sleep restriction on memory performance was observed only on the second testing session (which did not have interference), for the pre-training sleep restriction group, and for the semantically congruent faces. For the post-training sleep restriction group, although there were trends, the relevant effects did not reach significance. As discussed by the authors, this effect seems to result from a complex interplay between the sleep manipulation, the semantic congruence of the stimuli, and the disruptive effect of the interference task on the brain processes responsible for memory consolidation.

Four studies did not find any effects of sleep on face recognition memory [80,95,96,97]. After analyzing various central methodological aspects, such as how the sleep manipulation was achieved (between- or within-subjects), whether the sleep restriction was introduced pre- or post-encoding, the amount and time interval of the time spent awake (during the day or during the night), the type of task, the time of day of encoding and testing, and the dependent variables that were analyzed, we did not find a consistent pattern (see Table 2 for a summary). Thus, the absence of effects could be justified by the combination of a variety of factors that are not possible to pinpoint at the moment. One possibility is the lack of statistical power, and this was not reported in the papers. For example, Sheth and colleagues [96] used seven groups with 16 participants in each, not always including all the groups in the analysis.

The same methodological aspects that were compared for the studies with null effects also varied considerably among the studies with positive effects. For example, regarding the experimental task used, although the most common method to probe memory was the old-new paradigm, there was still considerable diversity between studies, mostly at the level of encoding (e.g., individual stimuli presentation with explicit memorization instructions [77]; training on a six-face array [90]; incidental learning of the faces during 3D virtual reality interaction [78]; indication of the emotional valence of faces [79]). During recognition, studies generally employed the classical old-new decision, with seen faces shown among new faces [77], even if sometimes an additional step was required (e.g., a temporal memory decision [88]). Considering this variability, in fact, a problem that seems important to point out from the literature on this topic is the lack of replication. Although 19 studies were included in the present scoping review, hinting that there has been some interest in the topic, most of them vary considerably in methodological options and it is hard to advocate for a true reproducibility of the reported effects. The only attempt at replication that we can identify is that of Mograss and collaborators, which conducted a series of experiments, using a similar task, while introducing small modifications in the protocol [77,81,92,93]. The results show consistency, with all studies with a within-subjects design report the positive effects of sleep on memory performance [77,92,93]. The only study showing discrepant results is the one using a between-subjects design [81], which explored individual differences in usual sleep length between groups, as has been discussed above. Therefore, it would be important to invest some effort in replication studies, which would help to consolidate the conclusions that can be drawn.

Another aspect that seems worth pointing out is the different methodological options regarding the manipulation of sleep vs. wake and when these periods occur. Some studies choose to introduce the sleep deprivation period during the night, i.e., participants either spend the full night awake [93], have sleep restricted to only a few hours [95], or have sleep systematically disturbed and interrupted [87], and this is compared to performance after a regular night of sleep. In other studies, the “no sleep” condition is simply an amount of time spent awake, typically during the day, compared to the same amount of time that includes sleep, typically during the night [77,91]. Although most authors conclude that better performance after sleep is due to the beneficial effect of sleep on memory consolidation in both conditions, this might not be the case, as limiting or eliminating sleep during the night disrupts the habitual circadian pattern, whereas an equal amount of time spent awake during the day fits that pattern. In this case, it is possible that a worse result after the wake period compared to the sleep period argues more in favor of memory deterioration due to a disruptive effect of the intervening wake time, instead of a beneficial effect of sleep per se. This was precisely the conclusion reached by Sheth et al. [96], who systematically varied acquisition time, test time, and retention interval. 

This also raises concerns regarding experiments that compare awake and sleep conditions that occur at different times of day (wake time during the day and sleep during the night), as this commonly involves differences between conditions for encoding and testing times. It has been established that various aspects of cognitive performance, including memory and attention, oscillate along the day [98,99] and varying testing times systematically with the experimental sleep-wake manipulation can represent a potential confound when interpreting results from these studies. Additionally, individual differences regarding chronotype, which represents the individual’s preference for the morning or evening period, has also been shown to influence cognitive performance [102]. Although some of the studies included in this review took this into consideration, e.g., [80,89], this was not the case for all the studies. As already mentioned, individual differences in chronobiological variables can introduce a significant amount of noise in the results and should be either controlled or considered in the data analysis of future studies.

Another variable that was not considered in the reviewed studies was individual face recognition ability. There is a growing body of evidence which demonstrates that people without any type of brain impairment vary considerably in their ability to recognize faces [103]. These naturally occurring individual differences should also be considered in these studies, particularly when between-subjects designs are adopted. Additionally, the interplay between individual differences in face recognition and sleep patterns should be explored. 

## 5. Conclusions

Regarding the first objective defined for the present scoping review, overall, the reviewed literature seems to support the positive impacts of sleep, as well as the detrimental effect of sleep restriction on face recognition memory. Nonetheless, the various studies are not unanimous, and there is substantial heterogeneity in the results reported (regarding both the presence and direction of effects, as well as the metrics and variables that show significant effects), and consequently in their conclusions regarding the specific effects of sleep on memory for faces. Therefore, further research is needed to systematically explore several potential methodological confounds that were identified in the present work and that may obscure the interpretation of sleep effects.

Thus, regarding the second objective of the scoping review, we suggest that future studies should focus primarily on controlling or systematically varying the times of day for learning and testing, the retention interval, the task used to test face recognition memory, and the sleep restriction protocol. Moreover, we believe that within-subjects designs should be employed whenever possible, to ensure a more adequate control of the potential impact of individual differences, such as chronotype, sleep duration, and face recognition ability, on the experimental outcomes. 

It should be noted that the aforementioned recommendation is not intended to discourage the use of new variables, the exploration of new interactions, or the use of new methodological manipulations. However, methodological homogenization is needed to perform an ultimate test of reproducibility, in order to consolidate the detrimental effect of sleep restriction on face recognition memory or not.

## Figures and Tables

**Table 1:** Summary of sample and experiment characteristics for each study included in the review.

First Author	Year	Sample Size	Age Mean (SD) [and/or Range]	Design *(for the Sleep Manipulation)*	Face Memory Task	Training/Encoding ToD	Testing/Recall ToD	Retention Interval	Prior Sleep Controlled? How?	Sleep Manipulation Control	Sleep Length	VDs	Main Results	Direction of Effect and Conclusion
*POSITIVE EFFECTS, i.e., Beneficial Effects of Sleep on Performance*														
**Barton and Pietrowsky** **[86]**	**2019**	40 [22F + 18M] sleep group (SG) = 20 [8F + 12M]; wake group (WG) = 20 [14F + 6M]	SG: 25.05 (6.88), WG: 27.05 (6.82)	**Between**-subjects; 2 groups: SG (1 h nap during retention) vs. WG (awake during retention)	**Old-new** recognition task, with emotion faces; explicit memorization instructions at encoding	12 pm or early afternoon	Afternoon (1–4 p.m.)	1 h	Sleep diaries (day before), abstain from substance intake	Polysomnography (lab)	No sleep past 8 a.m. the night before; SG: 1 h nap during retention WG: no nap during retention	Memory accuracy [Pr = hit rate-false alarm rate]; response bias [Br = false alarm rate/(1 − Pr)]; RTs	No significant main effects of group, or interactions with group, for memory accuracy, response bias and RT; positive correlation between N3 sleep and negative face recognition	No direct effect of sleep on face memory; correlation with N3 sleep suggests more elaborate processing during SWS and subsequently better face recognition for negative faces
**Frings** **[87]**	**2015**	182 (36F + 146M) Alert = 103, Fatigued = 79	[18,19,20,21,22,23,24]	**Between**-subjects; 2 groups: **alert** vs. **fatigued** *(subdivided in individual participants and teams, but not relevant for review purposes)*	**Target detection** task: two faces were initially shown and had to be subsequently identified among several sequentially presented quartets of faces, either target-present or target-absent	Alert: 7 p.m.; fatigued: 10–11 a.m.	Same as encoding	Immediate	No, but participants were instructed to be well rested before	Observational (field); Manipulation check with the Piper Fatigue Scale (confirmed)	**Alert**: prior sleep not controlled; **fatigued**: sleep duration <5 h for 2 consecutive nights, and systematically disturbed sleep	Hits, false alarms, sensitivity (*A*′), and response bias (*B*″)	Fatigued individuals had a higher response bias (i.e., a higher tendency towards identifying a target as absent) than alert individuals	No differences in detection ability. **Sleep disruption interfered with face recognition**, through response bias: sleep-disrupted individuals were more biased towards identifying the target as being absent (i.e., missing its recognition) than alert individuals.
**Harrison and Horne** **[88]**	**2000**	40 (20F + 20M)	23.4 [18,19,20,21,22,23,24,25,26,27,28,29,30,31,32,33,34]	**Between**-subjects; 4 groups: non-sleep-deprived (SD) with placed, non-SD with caffeine, SD with placebo, SD with caffeine. Total sleep deprivation for 35 h.	**Old-new** recognition task; two sequential sets of faces presented with a 5 min filler task between them; testing after a second 5 min. filler task. Assessed recognition (old-new?) and temporal memory (face belonged to 1st or 2nd set?).	Between 7:30 p.m. and 8:30 p.m.	Same as encoding	Almost immediate: 5 min between the two sets of 12 faces + 5 min after the second set, before testing	Actimetry and sleep diaries (3 nights before), controlled substance intake, normal sleepiness range and sleep habits (self-reported)	Non-SD groups: Actigraphy (home) SD groups: Observational (lab)	Non-SD groups: regular sleep duration; SD groups: no sleep	Recognition accuracy (*d*′), recency discrimination (*z* sensitivity), confidence for recency discrimination	No sig. effects of sleep condition on recognition; sig. effect of sleep condition on recency discrimination (SD worse) and confidence rating (SD groups were more confident about being correct when they were wrong)	**No-sleep effects** on face recognition accuracy, **but** a lack of sleep significantly **impaired** the **temporal memory** component for the recognition of faces
**Hussain et al.** **[89]**	**2008**	103 (74F + 29M) (G1-4: 24 each, G5: 7)	20.9 (3.31)	**Between**-subjects (for the sleep manipulation); 5 groups, with varying interval and sleep between sessions 1 and 2: G1 = 9 a.m. encoding—24 h interval with sleep; G2 = 9 p.m. encoding—24 h interval with sleep; G3 = 9 p.m. encoding—12 h interval with sleep; G4 = 9 a.m. encoding—12 h interval no sleep; G5 = 9 a.m. encoding—3 h interval no sleep	Explicit **face identification** task where a face that was presented at the beginning of each trial had to be identified amongst an array of 10 simultaneously presented faces; same stimuli in sessions 1 and 2	Two sessions for each group: G1 = 9 a.m. and 9 a.m. (next day); G2 = 9 p.m. and 9 p.m. (next day); G3 = 9 p.m. and 9 a.m. (next day); G4 = 9 a.m. and 9 p.m. (same day); G5 = 9 a.m. and 12 p.m. (same day)	Same as training ToD	Immediate	No, but chronotype was assessed (participants were unbiased towards ToD and values did not differ between groups)	None; **Sleep** groups: instructed to sleep normally overnight (between sessions); **No-Sleep** groups: instructed to not sleep or nap between sessions	Not controlled	Proportion of correct responses	Marginally greater improvement from session 1 to 2 (learning) in the sleep than no-sleep groups; small drop in performance of no-sleep groups in session 2, but restricted to first 102 trials; larger improvement in the 12 h sleep groups than in the 24 h sleep groups	**Small positive effect** of sleep on face identification, but the authors conclude that the effect of sleep on between session learning is negligible and that “robust perceptual learning for a face identification task can be obtained in the absence of sleep” (p. 2792)
**Martella et al.** **[90]**	**2012**	60 (34F + 26M) (Total sleep deprivation group = 26; control/sleep group = 34)	21.67 (5.54)	**Between**-subjects; 2 groups: sleep deprivation (**SD**) group vs. control/sleep group (**CG**)	Adapted **old-new** recognition task: training on a 6-face array, followed by individual faces and asked to indicate whether each face was in the previous array	**CG**: 8 p.m. **SD**: 4 a.m.	Same as encoding	Almost immediate: 5, 10, 25, or 32 s. random intervals	Daily sleep questionnaire (1 week), normal sleep duration and schedule	Observational (staying in the lab)	**CG**: unrestricted regular sleep schedule and duration; **SD**: no sleep	% correct responses; median correct RTs	Accuracy sig. higher for CG than SD; no effects for RTs	**Detrimental effect of sleep deprivation** on face recognition memory
**Maurer et al.** **[91]**	**2015**	22 (11F + 11M) **Exp. 1**: 14 (8F + 6M) **Exp. 2**: 8 (3F + 5M) *(data from 2 studies pooled for analysis)*	**Exp. 1:** 23.33 [21,22,23,24,25,26,27,28] **Exp. 2:** 24.75 (3.37) [22,23,24,25,26,27,28,29,30]	**Within**-subjects; 2 conditions: **sleep** vs. **wake** In **Exp. 2**, only **sleep** condition	**Face-name** task (explicit): learn to associate names to faces and later required to indicate if a specific pairing is correct or incorrect + rate confidence in the response (scale 1–9)	**Sleep** condition: evening, 2.5 h prior to scheduled sleep; **wake** condition: morning, 1.5 h after waking	**Sleep** condition: morning, 1.5 h after scheduled wake time; **wake** condition: evening, 12 h later, during the same wake episode	12 h	Actigraphy, sleep-wake diaries, kept a regular 8 h sleep schedule (1 week)	In the lab: 13-day inpatient circadian rhythm study; polysomnography during some sleep episodes	Approx. 8 h in the sleep condition	Proportion of correct responses; confidence rating; high confidence (rating 7–9) correct responses; RTs	Sig. higher proportion of correct responses in the sleep than the wake condition; 12% more highly confident responses in the sleep condition; 30% less incorrect responses in the sleep condition; no sig. effects for RTs	**Post-learning sleep** (in the retention period) had a significantly **positive effect** on recognition memory for face-name associations
**Mograss et al.** **[77]**	**2006**	13 (7F + 6M)	[21,22,23,24,25,26,27,28,29,30,31,32,33,34,35,36,37,38,39]	**Within**-subjects; 2 conditions: **sleep** between learn and test vs. **wake** between learn and test; 2 sessions, 3–7 days apart	**Old-new** recognition task; explicit memory task	**Sleep** condition: acquisition 5–7 p.m.; **wake** condition: acquisition 7–9 a.m.	**Sleep** condition: test/recall 7–9 a.m.; **wake** condition: test/recall 5–7 p.m.	**Sleep** condition: 12–16 h; **wake** condition: 8–12 h	Sleep agenda with questions about sleep habits and sleep quality for 3 days prior and during the experiment; Stanford Sleepiness Scale	Self-report questionnaires; observational (night of sleep spent in the lab)	Between 7.0 and 7.8 h/night on the various measures of sleep; no significant differences between sleep at home and in the lab	% of hits; RTs of correct responses	Significantly lower recognition of “old” items in the wake than in the sleep condition; no-sleep effects on RTs	**Better** performance after a night of sleep compared with daytime wake
**Mograss et al.** **[92]**	**2008**	18 (9F + 9M)	29 [18,19,20,21,22,23,24,25,26,27,28,29,30,31,32,33,34,35,36,37,38,39]	**Within**-subjects; 2 conditions: **sleep** between learn and test vs. **wake** between learn and test; 2 sessions, 4–7 days apart	**Old-new** recognition task; explicit memory task	**Sleep** condition: acquisition 4–6 pm; **wake** condition: acquisition 7–9 a.m.	**Sleep** condition: test/recall 7–9 a.m.; **wake** condition: test/recall 4–6 pm	**Sleep** condition: 13–17 h; **wake** condition: 7–11 h	Participants asked to keep regular sleep cycles for at least 3 days prior to the experiment, fill sleep diaries; refrain from taking naps during the day of testing	Sleep agenda; night of sleep in the lab (sleep condition) Stanford Sleepiness Scale (SSS) prior to the testing	Between 7.0 and 7.8 h/night on the various measures of sleep; no significant differences between sleep at home and in the lab	% of hits; % of misses; RTs of correct responses	Marginally more hits and significantly fewer misses to “old” stimuli in the sleep compared to the wake condition; no-sleep effects on RTs	**More accurate** performance after sleep compared to wakefulness during the retention period, suggesting a **positive** role of sleep in memory consolidation; fewer misses suggest that less information is forgotten after sleep
**Mograss et al.** **[93]**	**2009**	18 (9F + 9M)	21.9 (2.8) [18,19,20,21,22,23,24,25,26,27,28,29]	**Within**-subjects; 2 conditions: **sleep** vs. total sleep deprivation (**TSD**)	**Old-new** recognition task; explicit memory task	4–6 p.m.	7–9 a.m.	13–17 h	Participants asked to keep regular sleep cycles for at least 3 days prior to the experiment, fill sleep diaries; refrain from taking naps during the day of testing	Observational (two nights spent in the lab); Sleep quality questionnaire; vigilance scale	**Sleep** session: 7.6 h (0.32) **TSD** session: no sleep	% of hits; % of misses; RTs of correct responses and RTs of errors analyzed separately	Trend towards higher correct rejection of new stimuli following sleep compared to TSD (*p* = 0.07); sig. slower RTs on false alarms (FAs) after TSD compared to sleep	General tendency towards **worse performance** (increased difficulty to discriminate old and new items) **following TSD**, compared to normal sleep
**Solomonova et al.** **[78]**	**2017**	14 (Gender distribution not reported) REMD = 7 CG = 7	Not reported	**Between**-subjects; 2 groups: Partial REM sleep deprivation (**REMD**) group vs. control group (**CG**)	**Old-new** recognition task; incidental learning task: learning occurred through VR interactions with 3D characters (not real faces)	Evening (before bed time)	Morning	Not reported (one night)	Not reported	Observational (in the lab); EEG, electrooculogram	Mean = 5.41 h (groups differed on the amount of REM sleep, but not in the total sleep duration)	Proportion of correct responses	REMD performed significantly worse than CG; differential relationship between face recognition and fast and slow sleep spindles	**REM-deprived** participants showed **worse recognition** than non-REM-deprived participants; relationship with sleep spindles still unclear
**Wagner et al.** **[85]**	**2003** **(Main experiment)**	24 (all male)	Not reported	Mixed design: **within**-subjects manipulation: relevant retention interval early sleep (SWS) vs. late sleep (REM); **between**-subjects manipulation: sleeping or being wake during the retention interval	**Repetition priming** task (**implicit** task): during study, participants indicated the sex of the faces; during test, with the same faces among distractors, participants indicated the viewing direction of the faces	Early-sleep (**ES**) condition: 10:30 p.m. Late-sleep (**LS**) condition: 2:15–2:45 a.m.	Early-sleep (**ES**) condition: 2:15–2:45 a.m. Late-sleep (**LS**) condition: 6:00–6:30 a.m.	3 h	Healthy, regular sleepers, no smokers, medication-free	Polysomnography	**Sleep groups** ES: 3 h between learning and testing; LS: 3 h prior to learning, plus 3 h between learning and testing. **Wake groups** ES: no sleep; LS: 3 h prior to learning, no sleep between learning and testing	RTs of correct responses; priming effect calculated as the difference between the mean RT for ‘new’ and ‘old’ faces	No main effect of sleep-wake; significant interaction sleep-wake x ES/LS: increased RT for old faces compared to new faces (i.e., an inverse priming effect) in the LS condition	No main effects of sleep; however, REM sleep apparently impaired the expected priming effect. A possible explanation was that, instead of meaning impaired face memory, the inverse priming could indicate **improved recognition**, which was confirmed in the supp. experiment.
**Wagner et al.** **[85]**	**2003** **(Supplementary experiment)**	19 (all male)	Not reported	**Between**-subjects: relevant retention interval (early sleep (SWS) vs. late sleep (REM))	**Repetition priming** with explicit recognition task: study—indicates the sex of the faces; test—old-new recognition task	Early-sleep (**ES**) condition: 10:30 p.m. Late-sleep (**LS**) condition: 2:15–2:45 a.m.	Early-sleep (**ES**) condition: 2:15–2:45 a.m. Late-sleep (**LS**) condition: 6:00–6:30 a.m.	3 h	Healthy, regular sleepers, no smokers, medication-free	Polysomnography	ES: 3 h between learning and testing; LS: 3 h prior to learning, plus 3 h between learning and testing.	RTs of correct responses; priming effect calculated as the difference between the mean RT for ‘new’ and ‘old’ faces	VD: RTs significant positive priming effect, i.e., faster RT for old faces, more pronounced in the LS condition	**Positive effect of REM sleep** on memory, which strengthened automatic face recognition (this interfered with the implicit task used in the main experiment)
**Wagner et al.** **[79]**	**2007**	12 (5F + 7M)	[19,20,21,22,23,24,25,26,27,28,29,30]	**Within**-subjects; 2 conditions: **sleep** in the night following learning vs. **wake** in the night following learning; 2 sessions, at least 2 weeks apart	**Old-new** recognition task, with emotion faces; at encoding, participants had to indicate the emotional valence of the expression; no explicit memorization instruction	10:30–11:00 p.m.	6:00–6:30 p.m. (on the second evening after learning, allowing a full night of sleep at home)	Approx. 43 h	Regular sleep habits and normal sleep duration, night of adaptation in the lab	Night after encoding spent in the lab (asleep or awake); Polysomnography in the sleep condition; daytime sleep not allowed, and controlled by actigraphy in the wake condition	**Sleep** condition: approx. 8 h after learning, plus 1 night before testing; **wake** condition: total sleep deprivation after learning, plus 1 night before testing	Hit rate (HR); false alarm rate (FAR); memory accuracy, Pr [= HR - FAR]; response bias, Br [= FAR/(1 − Pr)]; RTs	Memory accuracy (hit rate-FA rate) was enhanced by sleep compared to wakefulness; sleep did not affect hits, false alarms, response bias, and RTs. **Sleep** condition: total sleep duration and amount of non-REM sleep in the consolidation night was significantly correlated with memory accuracy.	**Sleep** during consolidation **improved** face recognition memory; **positive relation** between memory accuracy and non-REM sleep and total sleep (but not REM sleep) duration
***NEGATIVE EFFECTS, i.e., the detrimental effects of sleep on performance***														
**Alberca-Reina et al.** **[94]**	**2014**	60 (31F + 29M)	22 (2.7) [18,19,20,21,22,23,24,25,26,27]	**Between**-subjects; 3 groups: **CG**—8 h of sleep before and after training; **SR_Pre-T_**—only 4 h of sleep pre-training; **SR_Post-T_**—only 4 h of sleep post-training *(i.e., acute sleep restriction)*	Day 1 (6:30 p.m.): semantic-perceptual matching task; informed of subsequent memory test. Day 2 (9 a.m.): retroactive interference task. Day 2 (11:30 am): associative memory test to the initial face pairs. Day 3 (11:30 am): **associative memory test** without interference	6:30 pm day 1), following a familiarization procedure at 5 pm	11:30 a.m. (day 2), 11:30 a.m. (day 3)	17 h (day 2), 41 h since the initial encoding (day 3)	Sleep diaries (1 week before experiment), structured interview, abstain from substance intake	Observational (sleep in the lab) on the night before and the first night after training; slept at home on the second night	CG: 8 h (days 1–3) SR_Pre-T_: 4 h (day 1), 8 h (days 2–3) SR_Post-T_: 8 h (days 1 and 3), 4 h (day 2)	RTs to correctly recognized stimuli; *d*′; estimates of recollection and familiarity processes derived from the dual-process signal detection model (Yonelinas et al., 1998)	**Session 2**: In comparison with the CG, the SR_Pre-T_ showed enhanced recognition for semantically congruent faces and decreased for semantically incongruent faces; SR_Post-T_ - trend for enhanced memory for both semantically congruent and incongruent associations	The results seem to suggest that **sleep restriction** exerted a **beneficial effect** on associative recognition memory for face pairs
**Mograss et al.** **[81]**	**2010**	24 (12F + 12M) **Short sleepers** (**SSs**) = 8 (3F/5M). **Average sleepers** (**ASs**) = 9 (4F/5M). **Long sleepers** (**LSs**) = 7 (4F/3M).	[18,19,20,21,22,23,24,25,26,27,28,29,30,31,32,33,34,35,36,37,38,39] **SSs**: 30.3 (5.8); **ASs**: 23.1 (4.0); **LSs**: 21.7 (2.1)	Between-subjects 3 groups: SSs vs. ASs vs. LSs	**Old-new** recognition task; explicit memory task	4–6 pm	7–9 a.m.	13–17 h	Participants asked to keep regular sleep cycles for at least 3 days prior to the experiment, fill sleep diaries and diverse questionnaires, interview	Night of sleep in the lab; sleep questionnaire; 7–10 day sleep log	Home: SSs: 6.8 h (0.23) ASs: 8.1 h (0.45) LSs: 9.1 h (0.52) Lab: SSs: 6.9 h (1.2) ASs: 8.3 h (0.70) LSs: 8.9 h (0.39)	% hits; % false alarms; % misses; RTs to correct responses for “old” and “new” items separately	**% hits**: **SSs** significantly **better** than ASs and LSs; ASs and LSs did not differ; the retention of old items in LSs did not differ from chance, while SSs were sig higher; % of hits decreased with sleep duration in SSs, but it increased with sleep duration in ASs and LSs	**Longer sleep** duration yielded **worse recognition memory** compared to average and short sleep durations Individual differences in sleep duration might be related to individual differences in face recognition memory
***NULL EFFECTS, i.e., sleep manipulation has no effect on performance***														
**Alberca-Reina et al.** **[95]**	**2015**	40 (21F + 19M)	21.8 (2.7) [18,19,20,21,22,23,24,25,26,27]	**Between**-subjects; 2 groups: control group (**CG**): sleep 8 h a night pre-training; acute sleep restriction (**ASR**): sleep 4 h a night pre-training	Day 1 (6:30 pm): training—semantic-perceptual matching task; informed of subsequent memory test. Day 2 (9:30 a.m.): retroactive interference task. Day 2 (12:00 p.m.): **associative memory test** to the initial face pairs.	6:30 pm (Day 1)	12 pm (Day 2, after a full night of sleep)	17 h 30 min	Sleep diaries (1 week before experiment), structured interview, abstain from substance intake	Observational (sleep in the lab) on the nights before and after training	CG.: 8 h ASR: 4 h	Hit rate; false alarm rate; RTs; *d*′ (associative *d*′ and semantic *d*′)	No significant differences between groups in any memory indices and no interactions with the semantic congruence of the face pairs	**No effect of sleep** restriction on recognition memory for face-face associations
**Chee et al.** **[80]**	**2010**	26 (14F + 12M)	20.7 (1.9)	**Within**-subjects; 2 sessions: rested wakefulness (**RW**) vs. sleep deprivation (**SD**) (1 week apart)	**Old-new** recognition task; incidental learning task: encoding occurred during a selective attention task with 3 conditions (attend faces, scenes, or both)	**RW**: 8 a.m. (after a night of sleep); **SD**: 5 a.m. (after a whole day and night awake without napping)	Same as encoding (shortly after)	10–15 min. after training	Actigraphy for 2 weeks, only participants with good sleeping habits; no extreme chronotype, controlled substance intake (24 h)	**SD**: observational (lab); **RW**: actigraphy (home)	**RW**: not reported (regular sleep); **SD**: no sleep	Analysis only of valid trials with responses to target stimuli during the encoding phase; RTs; response accuracy with *A*′	The effect of sleep on face recognition accuracy was not significant (contrary to what happen for the recognition of scenes, which was sig. reduced in SD)	**No effect of sleep**: face recognition was not affected by sleep deprivation; the authors suggest that it may be a more automatic process
**Sheth et al.** **[96]**	**2009**	112 (55F + 57M) 7 groups (16 participants in each)	25.25	**Between**-subjects; 7 groups that varied on acquisition and test times, as well as the retention period, intervening sleep, and intervening wake	**Old-new** recognition task + Confidence rating; Explicit memory task; Stimuli were computer-generated faces	G1: 9 p.m. G2: 9 p.m. G3: 9 p.m. G4: 9 a.m. G5: 9 a.m. G6: 9 a.m. G7: 9 p.m.	G1: 9 a.m. G2: 9 a.m. G3: 9 p.m. G4: 9 a.m. G5: 9 p.m. G6: 9 a.m. G7: 9 p.m.	G1: 12 h G2: 36 h G3: 24 h G4: 24 h G5: 12 h G6: 5 min G7: 5 min	Not controlled, but participants were selected on the basis of a screening questionnaire for substance intake, sleep habits and sleep duration	Actigraphy with a limited number of participants; sleep diaries	G1: sleep G2: sleep x2 G3: sleep G4: sleep G5: awake G6: awake G7: awake Sleep consisted in 1 or 2 normal nights of sleep (around 7.5 h)	Memory accuracy (*d*′); response bias (*c*)	Effect of sleep on *d*′ was not significant; effect on response bias: intervening wake during retention rendered the subject less likely to report seeing a test face before	**No effect**: sleep during retention did not appear to improve face recognition memory; however, the intervening wake time seems to impair memory strength
**Stare et al.** **[97]**	**2018**	93 (62F + 29M + 2 unknown)	21.38 (5.01) [18,19,20,21,22,23,24,25,26,27,28,29,30,31,32,33,34,35,36,37,38,39,40,41,42,43,44,45,46,47]	**Between**-subjects (4 groups: wakefulness, sleep, morning control, evening control)	**Old-new** recognition task; answer given on a confidence scale of 1 (confident not seen) to 4 (confident seen). Incidental memory task.	9 a.m. for the wakefulness and the morning control group; 9 p.m. for the sleep and the evening control group	9 p.m. for the wakefulness and the evening control group; 9 a.m. for the sleep and the morning control group	12 h for the wakefulness and sleep groups; immediately for the morning and evening control groups	Screening/demographics form; Stanford Sleepiness Scale	Polysomnography for participants staying in the lab; oral instructions to sleep or not sleep to those not staying in the lab	Participants in the sleep group were asked to try to sleep for at least 6 h	Corrected recognition scores (hits-false alarms)	No main effect of group (*p* = 0.36). No correlation of memory for faces with time spent in SWS, N2 and REM, total sleep time, or sleepiness.	**No effect** of sleep on memory for faces

**Table 2:** Summary of methodological aspects of the studies that found null results for sleep effects.

		Sleep Manipulation	Sleep Restriction	Amount of Restriction	Task	Encoding	Testing	VD
Alberca-Reina et al. [95]	2015	Between	Pre-training	4 h of sleep, night	Associative memory with interference	Evening	Morning	Various (RT, hits, FA, *d*′, associative *d*′, semantic *d*′)
Chee et al. [80]	2010	Within	Pre-training	Total sleep deprivation, night	Old-new; incidental learning	Morning	Morning	RT, *A*′
Sheth et al. [96]	2009	Between	Post-encoding	Day time awake (12 h)	Old-new; explicit memory	Varied	Varied	*d*′, *c*
Stare [97]	2018	Between	Post-encoding	Day time awake (12 h)	Old-new; incidental learning	Morning (wake)/evening (sleep)	Evening (wake)/morning (sleep)	Corrected recognition scores (hits-false alarms)

## Data Availability

Not applicable.

## References

[1] Mallon L., Broman J.E., Hetta J. (2002). Sleep Complaints Predict Coronary Artery Disease Mortality in Males: A 12-Year Follow-up Study of a Middle-Aged Swedish Population. J. Intern. Med..

[2] Cardoso J., Almeida T.C., Ramos C., Sousa S., Brito J. (2021). Bidirectional Relationship between Perceived Stress and Insomnia Symptoms: The Role of Coping and Quality of Life. Sleep Biol. Rhythm..

[3] Jarrin D.C., Alvaro P.K., Bouchard M.-A., Jarrin S.D., Drake C.L., Morin C.M. (2018). Insomnia and Hypertension: A Systematic Review. Sleep Med. Rev..

[4] Palagini L., Bruno R.M., Gemignani A., Baglioni C., Ghiadoni L., Riemann D. (2013). Sleep Loss and Hypertension: A Systematic Review. Curr. Pharm. Des..

[5] Anothaisintawee T., Reutrakul S., Van Cauter E., Thakkinstian A. (2016). Sleep Disturbances Compared to Traditional Risk Factors for Diabetes Development: Systematic Review and Meta-Analysis. Sleep Med. Rev..

[6] Zhang Y., Lin Y., Zhang J., Li L., Liu X., Wang T., Gao Z. (2019). Association between Insomnia and Type 2 Diabetes Mellitus in Han Chinese Individuals in Shandong Province, China. Sleep Breath..

[7] Irwin M.R. (2015). Why Sleep Is Important for Health: A Psychoneuroimmunology Perspective. Annu. Rev. Psychol..

[8] Vgontzas A.N., Fernandez-Mendoza J., Liao D., Bixler E.O. (2013). Insomnia with Objective Short Sleep Duration: The Most Biologically Severe Phenotype of the Disorder. Sleep Med. Rev..

[9] Buysse D.J. (2004). Insomnia, Depression and Aging. Assessing Sleep and Mood Interactions in Older Adults. Geriatrics.

[10] Shaffery J., Hoffmann R., Armitage R. (2003). The Neurobiology of Depression: Perspectives from Animal and Human Sleep Studies. Neuroscientist.

[11] Dew M.A., Hoch C.C., Buysse D.J., Monk T.H., Begley A.E., Houck P.R., Hall M., Kupfer D.J., Reynolds C.F. (2003). Healthy Older Adults’ Sleep Predicts All-Cause Mortality at 4 to 19 Years of Follow-Up. Psychosom. Med..

[12] Kripke D.F., Garfinkel L., Wingard D.L., Klauber M.R., Marler M.R. (2002). Mortality Associated with Sleep Duration and Insomnia. Arch. Gen. Psychiatry.

[13] Kim E.-J., Dimsdale J.E. (2007). The Effect of Psychosocial Stress on Sleep: A Review of Polysomnographic Evidence. Behav. Sleep. Med..

[14] Gilley R.R. (2022). The Role of Sleep in Cognitive Function: The Value of a Good Night’s Rest. Clin. EEG Neurosci..

[15] Nieto M., Motos B., Navarro B., Jimeno M.V., Fernández-Aguilar L., Ros L., Ricarte J.J., Latorre J.M. (2022). Relation between Nighttime Sleep Duration and Executive Functioning in a Nonclinical Sample of Preschool Children. Scand. J. Psychol..

[16] De Bruin E.J., van Run C., Staaks J., Meijer A.M. (2017). Effects of Sleep Manipulation on Cognitive Functioning of Adolescents: A Systematic Review. Sleep Med. Rev..

[17] Cruz T., García L., Álvarez M.A., Manzanero A.L. (2022). Sleep Quality and Memory Function in Healthy Ageing. Neurol. Engl. Ed..

[18] Ferrie J.E., Shipley M.J., Akbaraly T.N., Marmot M.G., Kivimäki M., Singh-Manoux A. (2011). Change in Sleep Duration and Cognitive Function: Findings from the Whitehall II Study. Sleep.

[19] Kronholm E., Sallinen M., Suutama T., Sulkava R., Era P., Partonen T. (2009). Self-Reported Sleep Duration and Cognitive Functioning in the General Population. J. Sleep Res..

[20] Lin L.-H., Xu W.-Q., Wang S.-B., Hu Q., Zhang P., Huang J.-H., Ke Y.-F., Ding K.-R., Hou C.-L., Jia F.-J. (2022). U-Shaped Association between Sleep Duration and Subjective Cognitive Complaints in Chinese Elderly: A Cross-Sectional Study. BMC Psychiatry.

[21] Mellow M.L., Crozier A.J., Dumuid D., Wade A.T., Goldsworthy M.R., Dorrian J., Smith A.E. (2022). How Are Combinations of Physical Activity, Sedentary Behaviour and Sleep Related to Cognitive Function in Older Adults? A Systematic Review. Exp. Gerontol..

[22] Richards A., Inslicht S.S., Metzler T.J., Mohlenhoff B.S., Rao M.N., O’Donovan A., Neylan T.C. (2017). Sleep and Cognitive Performance from Teens to Old Age: More Is Not Better. Sleep.

[23] Burke T.M., Scheer F.A.J.L., Ronda J.M., Czeisler C.A., Wright K.P. (2015). Sleep Inertia, Sleep Homeostatic and Circadian Influences on Higher-Order Cognitive Functions. J. Sleep Res..

[24] Bernstein J.P.K., DeVito A., Calamia M. (2019). Subjectively and Objectively Measured Sleep Predict Differing Aspects of Cognitive Functioning in Adults. Arch. Clin. Neuropsychol..

[25] Roth T., Costa e Silva J.A., Chase M.H. (2001). Sleep and Cognitive (Memory) Function: Research and Clinical Perspectives. Sleep Med..

[26] Goldstein A.N., Walker M.P. (2014). The Role of Sleep in Emotional Brain Function. Annu. Rev. Clin. Psychol..

[27] Anderson C., Platten C.R. (2011). Sleep Deprivation Lowers Inhibition and Enhances Impulsivity to Negative Stimuli. Behav. Brain Res..

[28] Motomura Y., Kitamura S., Oba K., Terasawa Y., Enomoto M., Katayose Y., Hida A., Moriguchi Y., Higuchi S., Mishima K. (2013). Sleep Debt Elicits Negative Emotional Reaction through Diminished Amygdala-Anterior Cingulate Functional Connectivity. PLoS ONE.

[29] Prather A.A., Bogdan R., Ahmad R., Hariri P. (2013). Impact of Sleep Quality on Amygdala Reactivity, Negative Affect, and Perceived Stress. Psychosom. Med..

[30] Yoo S.-S., Gujar N., Hu P., Jolesz F.A., Walker M.P. (2007). The Human Emotional Brain without Sleep--a Prefrontal Amygdala Disconnect. Curr. Biol. CB.

[31] Schwarz J.F.A., Popp R., Haas J., Zulley J., Geisler P., Alpers G.W., Osterheider M., Eisenbarth H. (2013). Shortened Night Sleep Impairs Facial Responsiveness to Emotional Stimuli. Biol. Psychol..

[32] Van der Helm E., Gujar N., Walker M.P. (2010). Sleep Deprivation Impairs the Accurate Recognition of Human Emotions. Sleep.

[33] Guadagni V., Burles F., Ferrara M., Iaria G. (2014). The Effects of Sleep Deprivation on Emotional Empathy. J. Sleep Res..

[34] Walker M.P. (2009). The Role of Sleep in Cognition and Emotion. Ann. N. Y. Acad. Sci..

[35] Jenkins J.G., Dallenbach K.M. (1924). Obliviscence during Sleep and Waking. Am. J. Psychol..

[36] Menz M.M., Rihm J.S., Salari N., Born J., Kalisch R., Pape H.C., Marshall L., Büchel C. (2013). The Role of Sleep and Sleep Deprivation in Consolidating Fear Memories. NeuroImage.

[37] Seeck-Hirschner M., Baier P.C., Weinhold S.L., Dittmar M., Heiermann S., Aldenhoff J.B., Göder R. (2012). Declarative Memory Performance Is Associated with the Number of Sleep Spindles in Elderly Women. Am. J. Geriatr. Psychiatry.

[38] Rauchs G., Bertran F., Guillery-Girard B., Desgranges B., Kerrouche N., Denise P., Foret J., Eustache F. (2004). Consolidation of Strictly Episodic Memories Mainly Requires Rapid Eye Movement Sleep. Sleep.

[39] Diekelmann S., Wilhelm I., Born J. (2009). The Whats and Whens of Sleep-Dependent Memory Consolidation. Sleep Med. Rev..

[40] Ashworth A., Hill C.M., Karmiloff-Smith A., Dimitriou D. (2014). Sleep Enhances Memory Consolidation in Children. J. Sleep Res..

[41] Hoedlmoser K. (2020). Sleep and Memory in Children. Curr. Sleep Med. Rep..

[42] Palmer C.A., Alfano C.A. (2017). Sleep and Emotion Regulation: An Organizing, Integrative Review. Sleep Med. Rev..

[43] Tempesta D., De Gennaro L., Natale V., Ferrara M. (2015). Emotional Memory Processing Is Influenced by Sleep Quality. Sleep Med..

[44] Walker M.P., Stickgold R. (2006). Sleep, Memory, and Plasticity. Annu. Rev. Psychol..

[45] Ellenbogen J.M., Hu P.T., Payne J.D., Titone D., Walker M.P. (2007). Human Relational Memory Requires Time and Sleep. Proc. Natl. Acad. Sci. USA.

[46] Wagner U., Gais S., Haider H., Verleger R., Born J. (2004). Sleep Inspires Insight. Nature.

[47] Fortier-Brochu É., Beaulieu-Bonneau S., Ivers H., Morin C.M. (2012). Insomnia and Daytime Cognitive Performance: A Meta-Analysis. Sleep Med. Rev..

[48] Ackermann S., Rasch B. (2014). Differential Effects of Non-REM and REM Sleep on Memory Consolidation?. Curr. Neurol. Neurosci. Rep..

[49] Smith C. (2001). Sleep States and Memory Processes in Humans: Procedural versus Declarative Memory Systems. Sleep Med. Rev..

[50] Boyce R., Williams S., Adamantidis A. (2017). REM Sleep and Memory. Curr. Opin. Neurobiol..

[51] Siegel J.M. (2001). The REM Sleep-Memory Consolidation Hypothesis. Science.

[52] Vertes R.P., Eastman K.E. (2000). The Case against Memory Consolidation in REM Sleep. Behav. Brain Sci..

[53] Smith C., Macneill C. (1994). Impaired Motor Memory for a Pursuit Rotor Task Following Stage 2 Sleep Loss in College Students. J. Sleep Res..

[54] Wagner U., Gais S., Born J. (2001). Emotional Memory Formation Is Enhanced across Sleep Intervals with High Amounts of Rapid Eye Movement Sleep. Learn. Mem..

[55] MacDonald K.J., Cote K.A. (2021). Contributions of Post-Learning REM and NREM Sleep to Memory Retrieval. Sleep Med. Rev..

[56] White D., Kemp R.I., Jenkins R., Matheson M., Burton A.M. (2014). Passport Officers’ Errors in Face Matching. PLoS ONE.

[57] Bruce V., Young A. (1986). Understanding Face Recognition. Br. J. Psychol. Lond. Engl. 1953.

[58] Haxby J.V., Hoffman E.A., Gobbini M.I. (2000). The Distributed Human Neural System for Face Perception. Trends Cogn. Sci..

[59] Li J., He D., Zhou L., Zhao X., Zhao T., Zhang W., He X. (2019). The Effects of Facial Attractiveness and Familiarity on Facial Expression Recognition. Front. Psychol..

[60] Silva A., Macedo A.F., Albuquerque P.B., Arantes J. (2016). Always on My Mind? Recognition of Attractive Faces May Not Depend on Attention. Front. Psychol..

[61] Paulmann S., Pell M.D. (2009). Facial Expression Decoding as a Function of Emotional Meaning Status: ERP Evidence. Neuroreport.

[62] Santos I.M., Iglesias J., Olivares E.I., Young A.W. (2008). Differential Effects of Object-Based Attention on Evoked Potentials to Fearful and Disgusted Faces. Neuropsychologia.

[63] Santos I.M., Young A.W. (2011). Inferring Social Attributes from Different Face Regions: Evidence for Holistic Processing. Q. J. Exp. Psychol..

[64] Sutherland C.A.M., Oldmeadow J.A., Santos I.M., Towler J., Michael Burt D., Young A.W. (2013). Social Inferences from Faces: Ambient Images Generate a Three-Dimensional Model. Cognition.

[65] Wilmer J.B., Germine L., Chabris C.F., Chatterjee G., Williams M., Loken E., Nakayama K., Duchaine B. (2010). Human Face Recognition Ability Is Specific and Highly Heritable. Proc. Natl. Acad. Sci. USA.

[66] Kanwisher N., McDermott J., Chun M.M. (1997). The Fusiform Face Area: A Module in Human Extrastriate Cortex Specialized for Face Perception. J. Neurosci..

[67] Gosling A., Eimer M. (2011). An Event-Related Brain Potential Study of Explicit Face Recognition. Neuropsychologia.

[68] Avidan G., Behrmann M. (2008). Implicit Familiarity Processing in Congenital Prosopagnosia. J. Neuropsychol..

[69] Diekelmann S., Born J. (2010). The Memory Function of Sleep. Nat. Rev. Neurosci..

[70] Stickgold R. (2005). Sleep-Dependent Memory Consolidation. Nature.

[71] Beattie L. (2018). How Does Sleep Affect the Perception of Facial Emotion?. Sleep.

[72] Beattie L., Walsh D., McLaren J., Biello S.M., White D. (2016). Perceptual Impairment in Face Identification with Poor Sleep. R. Soc. Open Sci..

[73] Crönlein T., Langguth B., Eichhammer P., Busch V. (2016). Impaired Recognition of Facially Expressed Emotions in Different Groups of Patients with Sleep Disorders. PLoS ONE.

[74] Holding B.C., Sundelin T., Cairns P., Perrett D.I., Axelsson J. (2019). The Effect of Sleep Deprivation on Objective and Subjective Measures of Facial Appearance. J. Sleep Res..

[75] De Almondes K.M., Júnior F.W.N.H., Leonardo M.E.M., Alves N.T. (2020). Facial Emotion Recognition and Executive Functions in Insomnia Disorder: An Exploratory Study. Front. Psychol..

[76] Zhang J., Chan A.B., Lau E.Y.Y., Hsiao J.H. (2019). Individuals with Insomnia Misrecognize Angry Faces as Fearful Faces While Missing the Eyes: An Eye-Tracking Study. Sleep.

[77] Mograss M., Godbout R., Guillem F. (2006). The ERP Old-New Effect: A Useful Indicator in Studying the Effects of Sleep on Memory Retrieval Processes. Sleep.

[78] Solomonova E., Stenstrom P., Schon E., Duquette A., Dubé S., O’Reilly C., Nielsen T. (2017). Sleep-Dependent Consolidation of Face Recognition and Its Relationship to REM Sleep Duration, REM Density and Stage 2 Sleep Spindles. J. Sleep Res..

[79] Wagner U., Kashyap N., Diekelmann S., Born J. (2007). The Impact of Post-Learning Sleep vs. Wakefulness on Recognition Memory for Faces with Different Facial Expressions. Neurobiol. Learn. Mem..

[80] Chee M.W.L., Tan J.C., Parimal S., Zagorodnov V. (2010). Sleep Deprivation and Its Effects on Object-Selective Attention. NeuroImage.

[81] Mograss M.A., Guillem F., Stickgold R. (2010). Individual Differences in Face Recognition Memory: Comparison among Habitual Short, Average, and Long Sleepers. Behav. Brain Res..

[82] Peters M.D.J., Marnie C., Tricco A.C., Pollock D., Munn Z., Alexander L., McInerney P., Godfrey C.M., Khalil H. (2020). Updated Methodological Guidance for the Conduct of Scoping Reviews. JBI Evid. Synth..

[83] Tricco A.C., Lillie E., Zarin W., O’Brien K.K., Colquhoun H., Levac D., Moher D., Peters M.D.J., Horsley T., Weeks L. (2018). PRISMA Extension for Scoping Reviews (PRISMA-ScR): Checklist and Explanation. Ann. Intern. Med..

[84] Ouzzani M., Hammady H., Fedorowicz Z., Elmagarmid A. (2016). Rayyan—A Web and Mobile App for Systematic Reviews. Syst. Rev..

[85] Wagner U., Hallschmid M., Verleger R., Born J. (2003). Signs of REM Sleep Dependent Enhancement of Implicit Face Memory: A Repetition Priming Study. Biol. Psychol..

[86] Barton S., Pietrowsky R. (2019). Selective Enhancement of Processing of Negative Faces During Slow-Wave Sleep. Sleep Hypn..

[87] Frings D. (2015). The Effects of Low Levels of Fatigue on Face Recognition among Individuals and Team Members. J. Appl. Soc. Psychol..

[88] Harrison Y., Horne J.A. (2000). Sleep Loss and Temporal Memory. Q. J. Exp. Psychol..

[89] Hussain Z., Sekuler A.B., Bennett P.J. (2008). Robust Perceptual Learning of Faces in the Absence of Sleep. Vision Res..

[90] Martella D., Plaza V., Estévez A.F., Castillo A., Fuentes L.J. (2012). Minimizing Sleep Deprivation Effects in Healthy Adults by Differential Outcomes. Acta Psychol..

[91] Maurer L., Zitting K.-M., Elliott K., Czeisler C.A., Ronda J.M., Duffy J.F. (2015). A New Face of Sleep: The Impact of Post-Learning Sleep on Recognition Memory for Face-Name Associations. Neurobiol. Learn. Mem..

[92] Mograss M.A., Guillem F., Godbout R. (2008). Event-Related Potentials Differentiates the Processes Involved in the Effects of Sleep on Recognition Memory. Psychophysiology.

[93] Mograss M.A., Guillem F., Brazzini-Poisson V., Godbout R. (2009). The Effects of Total Sleep Deprivation on Recognition Memory Processes: A Study of Event-Related Potential. Neurobiol. Learn. Mem..

[94] Alberca-Reina E., Cantero J.L., Atienza M. (2014). Semantic Congruence Reverses Effects of Sleep Restriction on Associative Encoding. Neurobiol. Learn. Mem..

[95] Alberca-Reina E., Cantero J.L., Atienza M. (2015). Impact of Sleep Loss before Learning on Cortical Dynamics during Memory Retrieval. NeuroImage.

[96] Sheth B.R., Nguyen N., Janvelyan D. (2009). Does Sleep Really Influence Face Recognition Memory?. PLoS ONE.

[97] Stare C.J., Gruber M.J., Nadel L., Ranganath C., Gómez R.L. (2018). Curiosity-Driven Memory Enhancement Persists over Time but Does Not Benefit from Post-Learning Sleep. Cogn. Neurosci..

[98] Monteiro F., Rodrigues P., Nascimento C.S., Simões F., Miguel M. (2021). The Daily Rhythms of Working Memory and Their Methodological Constraints: A Critical Overview. Biol. Rhythm Res..

[99] Valdez P., Ramírez C., García A., Talamantes J., Armijo P., Borrani J. (2005). Circadian Rhythms in Components of Attention. Biol. Rhythm Res..

[100] Walker M.P. (2008). Cognitive Consequences of Sleep and Sleep Loss. Sleep Med..

[101] Pizinger T.M., Aggarwal B., St-Onge M.-P. (2018). Sleep Extension in Short Sleepers: An Evaluation of Feasibility and Effectiveness for Weight Management and Cardiometabolic Disease Prevention. Front. Endocrinol..

[102] Schmidt C., Collette F., Cajochen C., Peigneux P. (2007). A Time to Think: Circadian Rhythms in Human Cognition. Cogn. Neuropsychol..

[103] Wilmer J.B. (2017). Individual Differences in Face Recognition: A Decade of Discovery. Curr. Dir. Psychol. Sci..

